# Glycolytic inhibitor 2-deoxyglucose simultaneously targets cancer and endothelial cells to suppress neuroblastoma growth in mice

**DOI:** 10.1242/dmm.021667

**Published:** 2015-10-01

**Authors:** Chao-Cheng Huang, Shuo-Yu Wang, Li-Ling Lin, Pei-Wen Wang, Ting-Ya Chen, Wen-Ming Hsu, Tsu-Kung Lin, Chia-Wei Liou, Jiin-Haur Chuang

**Affiliations:** 1 Department of Pathology, Kaohsiung Chang Gung Memorial Hospital and Chang Gung University College of Medicine, Kaohsiung 833, Taiwan; 2 Biobank and Tissue Bank, Kaohsiung Chang Gung Memorial Hospital, Kaohsiung 833, Taiwan; 3 Department of Pediatrics, Chi-Mei Medical Center, Tainan 710, Taiwan; 4 Graduate Institute of Clinical Medical Sciences, Chang Gung University College of Medicine, Taoyuan 333, Taiwan; 5 Department of Medical Research, Kaohsiung Chang Gung Memorial Hospital, Kaohsiung 833, Taiwan; 6 The Mitochondrial Research Unit, Kaohsiung Chang Gung Memorial Hospital, Kaohsiung 833, Taiwan; 7 Department of Internal and Nuclear Medicine, Kaohsiung Chang Gung Memorial Hospital and Chang Gung University College of Medicine, Kaohsiung 833, Taiwan; 8 Department of Surgery, National Taiwan University Hospital, National Taiwan University College of Medicine, Taipei 100, Taiwan; 9 Department of Neurology, Kaohsiung Chang Gung Memorial Hospital and Chang Gung University College of Medicine, Kaohsiung 833, Taiwan; 10 The Division of Pediatric Surgery, Kaohsiung Chang Gung Memorial Hospital and Chang Gung University College of Medicine, Kaohsiung 833, Taiwan

**Keywords:** Neuroblastoma, 2-deoxyglucose, Xenograft, *MYCN* amplification, Endothelial cell

## Abstract

Neuroblastoma is characterized by a wide range of clinical manifestations and associated with poor prognosis when there is amplification of *MYCN* oncogene or high expression of Myc oncoproteins. In a previous *in vitro* study, we found that the glycolytic inhibitor 2-deoxyglucose (2DG) could suppress the growth of neuroblastoma cells, particularly in those with *MYCN* amplification. In this study, we established a mouse model of neuroblastoma xenografts with SK-N-DZ and SK-N-AS cells treated with 2DG by intraperitoneal injection twice a week for 3 weeks at 100 or 500 mg/kg body weight. We found that 2DG was effective in suppressing the growth of both *MYCN*-amplified SK-N-DZ and *MYCN*-non-amplified SK-N-AS neuroblastoma xenografts, which was associated with downregulation of HIF-1α, PDK1 and c-Myc, and a reduction in the number of tumor blood vessels. *In vitro* study showed that 2DG can suppress proliferation, cause apoptosis and reduce migration of murine endothelial cells, with inhibition of the formation of lamellipodia and filopodia and disorganization of F-actin filaments. The results suggest that 2DG might simultaneously target cancer cells and endothelial cells in the neuroblastoma xenografts in mice regardless of the status of *MYCN* amplification, providing a potential therapeutic opportunity to use 2DG or other glycolytic inhibitors for the treatment of patients with refractory neuroblastoma.

## Abstract (precis)

**Drug Discovery Collection:** Neuroblastomas are sensitive to treatment with the glycolytic inhibitor 2-deoxyglucose, which can simultaneously target cancer and endothelial cells to suppress tumor growth.

## INTRODUCTION

Growth of most solid tumors is metabolically active and highly dependent on blood vessels to supply nutrients and to remove metabolic waste. Metabolic reprogramming, including aerobic glycolysis, *de novo* lipid biosynthesis and glutamine-dependent anaplerosis, fuels cancer cell growth and proliferation (DeBerardinis et al., 2008). Diverse metabolic adaptations allow cancer cells to survive and thrive in harsh environments, and the metabolic landscape of the tumor should therefore be studied explicitly to treat the tumor and its microenvironment at the same time (Sousa and Kimmelman, 2014). However, cancer and endothelial metabolism have only recently been recognized to exist like brothers in arms, in that endothelial cells have been found to be highly glycolytic, exactly like cancer cells (De Bock et al., 2013a,b; Rivera and Bergers, 2014; Verdegem et al., 2014). The results of these studies offer novel opportunity to treat solid tumors by targeting cancer cells and endothelial cells simultaneously.

Neuroblastoma (NB) is a solid tumor in children characterized by a wide range of clinical manifestations and by a poor prognosis when there is amplification of *MYCN* oncogene or high expression of Myc oncoproteins (Haupt et al., 2010; Maris et al., 2007; Wang et al., 2013, 2015). Myc oncoproteins are deeply involved in metabolic regulation and proliferation of cancer cells (DeBerardinis et al., 2008; Osthus et al., 2000; Wise et al., 2008). SK-N-DZ is a *MYCN*-amplified NB cell line with overexpression of N-Myc oncoprotein, whereas SK-N-AS is a *MYCN*-non-amplified NB cell line reported to exhibit high expression of c-Myc (Hossain et al., 2013; Puissant et al., 2013; Toyoshima et al., 2012). The results of our previous *in vitro* study confirmed a role for the glycolytic inhibitor 2-deoxyglucose (2DG) in suppressing the growth of NB cells, particularly in those with *MYCN* amplification (Chuang et al., 2013). In this study, we report that 2DG is also effective to treat *MYCN*-non-amplified NB xenografts, as well as *MYCN*-amplified NB xenografts in mice. We found that simultaneous targeting of cancer cells and endothelial cells by 2DG *in vivo* was responsible for successful suppression of the growth of NB, regardless of the status of *MYCN* amplification.

## RESULTS

### Treatment with 2DG induces shrinkage of NB tumors in NOD/SCID mice

To study the effect of 2DG on NB xenografts, we measured the size and the weight of the tumor harvested from the right flank of NOD/SCID mice on the 27th day after the experiment. The tumors from the control DZ xenografts reached a considerable size, weighing 3.081±0.498 g. Treatment with 100 or 500 mg/kg body weight (hereafter, kg refers to body weight) of 2DG resulted in significant reduction of tumor weight to 0.590±0.193 and 0.503±0.235 g, respectively (both *P*<0.05). Likewise, on the 27th day after treatment, the tumors from the control AS xenografts was also large, weighing 1.839±0.451 g. Treatment of the mice with 100 and 500 mg/kg of 2DG resulted in a reduction of tumor weight to 0.647±0.276 g (*P*<0.05) and 1.228±0.458 g (*P*=0.160), respectively (supplementary material Fig. S1).

**TRANSLATIONAL IMPACT**

**Clinical issue**

Usually, solid tumor growth is metabolically active and highly dependent on blood vessels to supply nutrients and to remove metabolic waste, hence cancer cells need to acquire diverse metabolic adaptations and stimulate neovascularization to survive and thrive in harsh environments. Neuroblastoma is one of the most common pediatric solid tumors, with various clinical presentations. The prognosis is poor when the tumor is associated with amplification of *MYCN* oncogene or high expression of Myc oncoproteins, which are involved in the metabolic regulation of cancer cells. A previous *in vitro* study has shown that the glycolytic inhibitor 2-deoxyglucose (2DG) induces glucose deprivation and suppresses tumor cell growth in neuroblastoma, especially in those types with *MYCN* amplification. However, it was not clear whether 2DG inhibits angiogenesis in addition to directly killing tumor cells.

**Results**

The authors used a mouse model of neuroblastoma xenografts, in which human SK-N-DZ and SK-N-AS cells were transplanted into NOD/SCID mice. Mice were treated with 2DG by intraperitoneal injection to study the anti-tumor mechanisms of 2DG in neuroblastoma. The authors found that 2DG is able to suppress the tumor growth not only in *MYCN*-amplified SK-N-DZ xenografts but also in *MYCN*-non-amplified SK-N-AS xenografts. This anti-tumor effect was associated with downregulation of HIF-1α, PDK1 and c-Myc, and a reduction in the number of tumor vessels. They also showed that 2DG suppresses cell proliferation, induces apoptosis and reduces migration of murine endothelial cells SVEC4-10. In addition, by combining CellMask and F-actin staining with laser confocal microscopic examination, they reported inhibition of the formation of lamellipodia and filopodia and disorganization of F-actin filaments of SVEC4-10 cells after treatment with 2DG.

**Implications and future directions**

This study discloses the unexpected finding that in addition to its therapeutic effect on *MYCN*-amplified SK-N-DZ xenografts, the glycolytic inhibitor 2DG is also effective to reduce the growth of *MYCN*-non-amplified SK-N-AS xenografts in mice, which was not observed in the previous *in vitro* study. The finding that endothelial cells are also sensitive to 2DG treatment underscores the role of 2DG in the inhibition of tumor angiogenesis in neuroblastoma in addition to its ability to suppress tumor cells per se. The double therapeutic effect of 2DG in the treatment of mouse neuroblastoma xenografts suggests a strategy that could be useful to develop anti-cancer agents for other tumors.

### 2DG decreases the expression of HIF-1α, PDK1 and c-Myc, but not Bax or Bak in NB xenograft

**Fig. 1.**
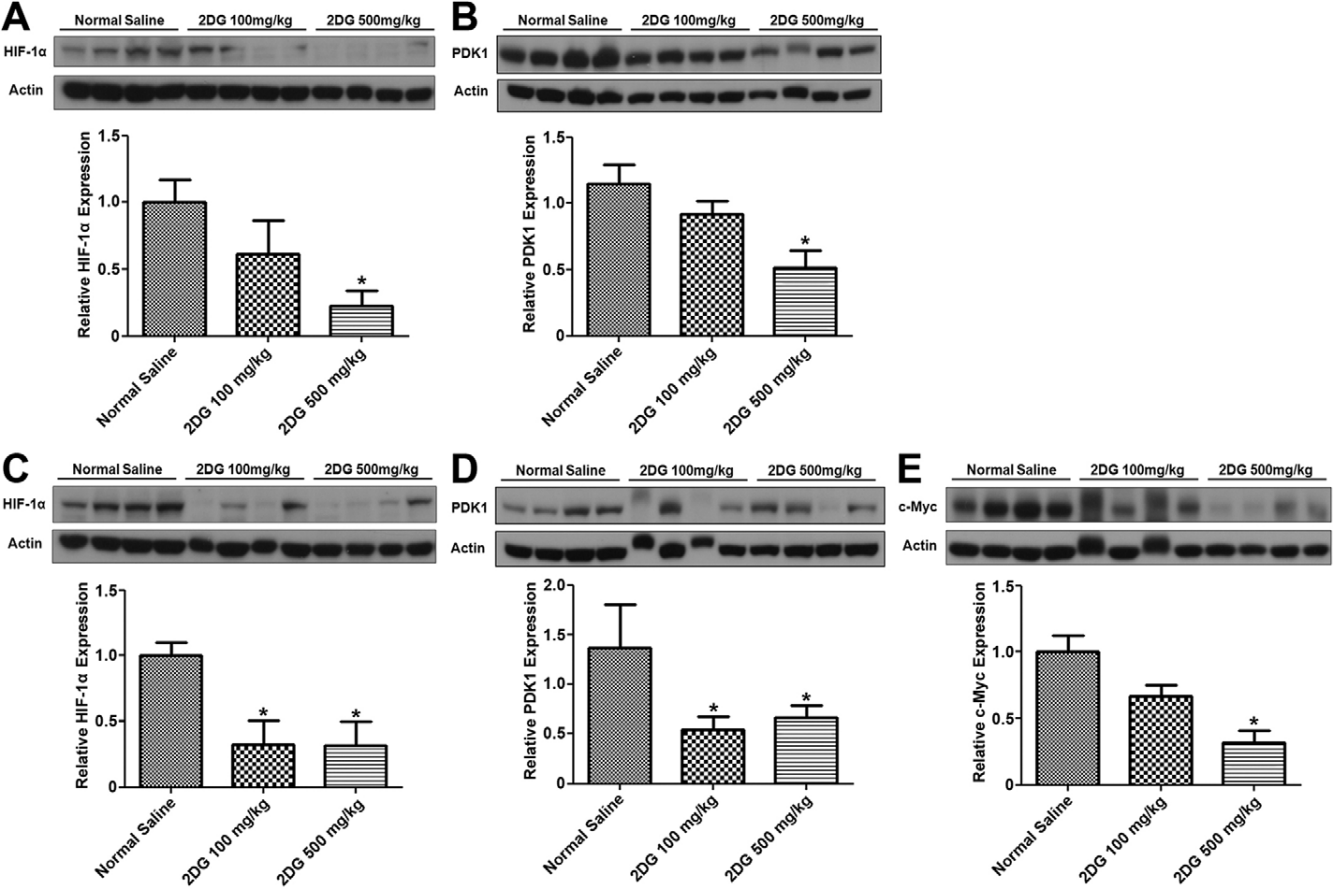
**2DG downregulates the expression of HIF-1α, PDK1 and c-Myc in NB xenograft.** The mice received subcutaneous injection of SK-N-DZ NB cells (A,B) and SK-N-AS NB cells (C-E) and received a total of six doses of normal saline or 2DG. The xenografts were harvested on the 27th day and then subjected to western blotting using the indicated antibodies. Graph data are the means±s.e.m.; *P<0.05. *n*=4 (A,C,E); *n*=7 for normal saline group and *n*=4 for 2DG groups (B); and *n*=7 (D).

To assess the *in vivo* effects of 2DG on HIF-1α, PDK1 and c-MYC expression in NB xenografts, western blotting of the tissue homogenates was performed. A significant reduction of HIF-1α and PDK1 was found in the tumors of DZ (Fig. 1A,B), as well as in those of AS xenograft (Fig. 1C,D) when treated with 100 and 500 mg/kg of 2DG, compared with the control. Interestingly, c-Myc expression was high in AS, and 2DG treatment also resulted in dose-dependent reduction of c-Myc, which was significant at the dose of 500 mg/kg (Fig. 1E). To our surprise, downregulation of HIF-1α, PDK1 and c-Myc did not result in a decrease of the pro-apoptotic proteins Bax or Bak in either DZ or AS xenograft (supplementary material Fig. S2A,B). A significant decrease of Bad in DZ was counteracted by a decrease of p-Bad. Likewise, a decrease of Bad in AS was associated with a significant decrease of p-Bad (supplementary material Fig. S2C,D).

### Immunohistochemical staining of PDK1 in NB xenografts

**Fig. 2.**
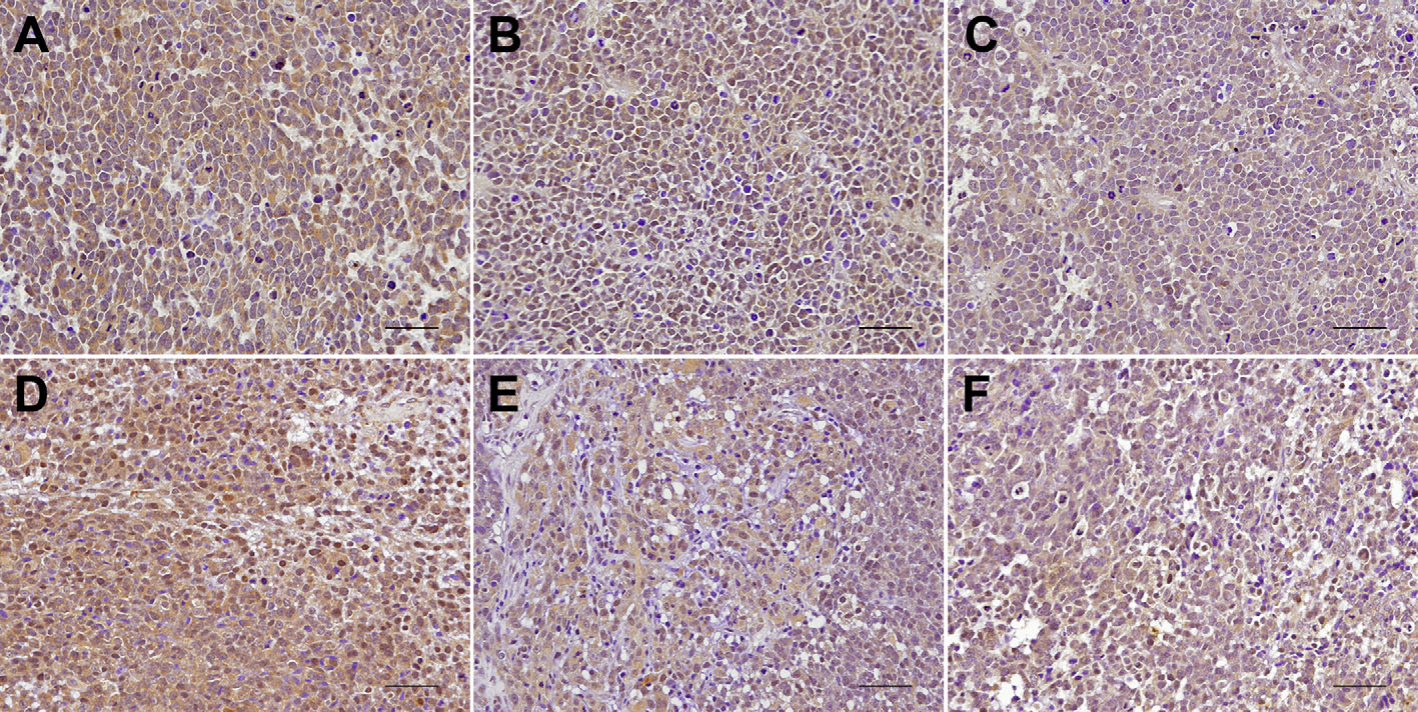
**Immunohistochemical staining for PDK1 expression in neuroblastoma xenografts.** Representative xenografts of SK-N-DZ NB cells (A-C) and SK-N-AS NB cells (D-F) receiving a total of six doses each of normal saline (A,D), 100 mg/kg of 2DG (B,E) or 500 mg/kg of 2DG (C,F) were immunostained with anti-PDK1 antibody after formalin fixation and paraffin embedding. PDK1 expression is present in both the nuclei and the cytoplasm of the tumor cells. Decreased PDK1 immunostaining is present with 2DG treatment. Scale bars, 50 μm.

To unveil the effect of 2DG treatment on PDK1 expression, immunohistochemical staining was also performed in NB xenografts. In both DZ and AS xenografts, PDK1 expression was observed in the nuclei and cytoplasm of the tumor cells (Fig. 2). A decrease in staining intensity was found after 2DG treatment, which was consistent with the findings by western blotting (Fig. 1B,D).

### 2DG significantly suppresses proliferation, causes apoptosis and reduces migration of murine endothelial cells

**Fig. 3.**
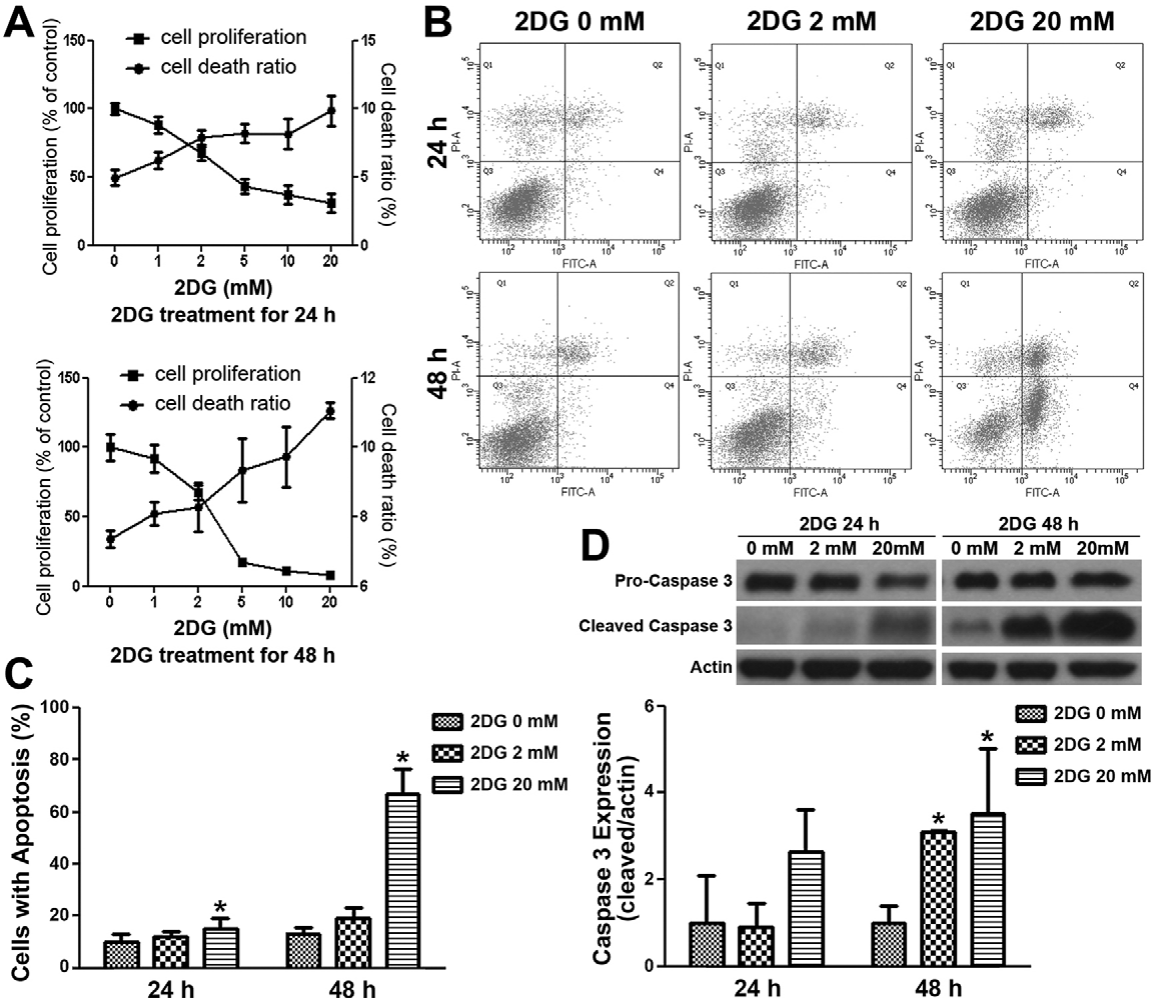
**2DG significantly suppresses cell proliferation and induces apoptosis in murine endothelial cells.** (A) SVEC4-10 cells were treated with six different dosages of 2DG for 24 or 48 h, and then a cell proliferation assay and Trypan Blue exclusion assay were conducted. (B) After 2DG treatment for 24 or 48 h, SVEC4-10 cells were stained with annexin V (fluorescein isothiocyanate)/PI for apoptosis analysis by flow cytometry. (C) The ratio of cells with apoptosis (annexin V^+^, PI^−^ and annexin V^+^, PI^+^) was measured. (D) Cleaved caspase-3 expression was measured by western blotting. Data are means±s.d.; *P<0.05, *n*=4 (A-C); *n*=3 (D).

Given that the *in vivo* effects of 2DG on the animal xenograft could not be explained simply by its action on NB cells, we studied the effects of 2DG on the mouse endothelial cell line SVEC4-10. A dose-dependent decrease of cell proliferation and increase of cell death (measured by Trypan Blue exclusion test) was found in SVEC4-10 cells treated with 2DG for 24 h and was more prominent at 48 h (Fig. 3A). Further analysis, by using annexin V and propidium iodide (PI) staining followed by flow cytometry, revealed a dose-dependent increase in SVEC4-10 cell apoptosis after treatment with 2DG for 24 h, which was more prominent at 48 h (Fig. 3B,C). Furthermore, we also found a dose-dependent increase of cleaved caspase-3 expression, which was significant at 24 and 48 h after treatment with 2DG (Fig. 3D).

**Fig. 4.**
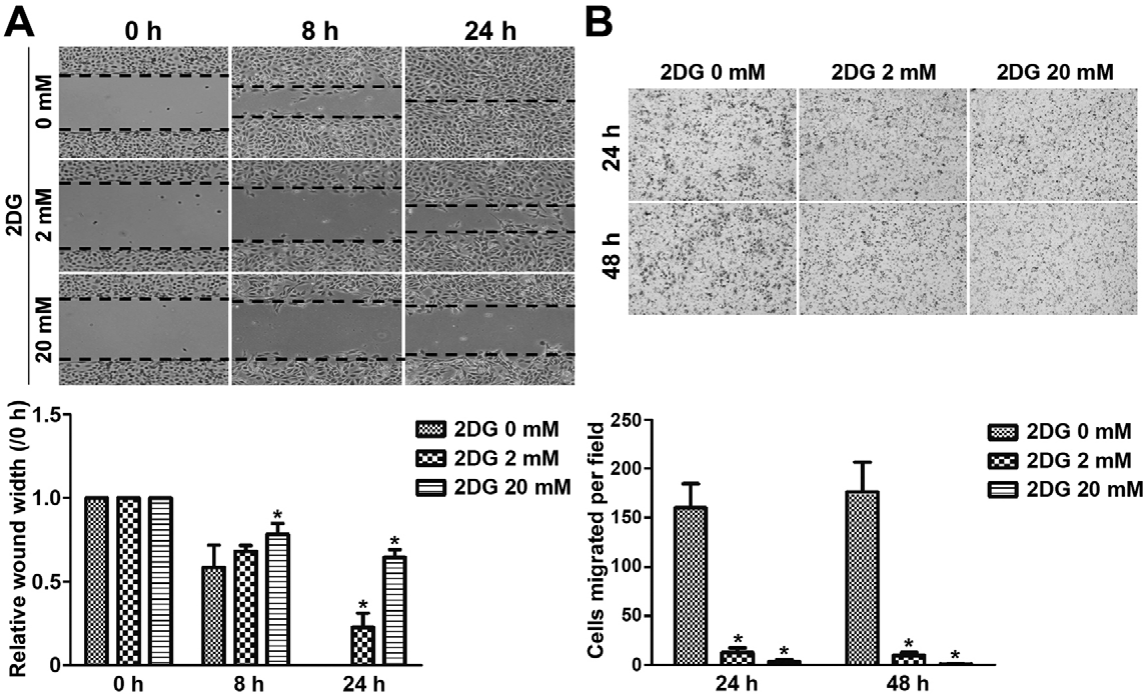
**2DG decreases wound closure and inhibits cell migration in murine endothelial cells.** (A) SVEC4-10 cells treated with 2DG fill the wound area (area between the two dotted lines) more slowly than those untreated at 8 and 24 h. The wound-healing assay was expressed as relative wound width (8 or 24 h average wound width divided by 0 h wound width). (B) For the migration assay, SVEC4-10 cells were cultured in a Boyden chamber and treated with 2DG. After 24 and 48 h, cells were fixed and stained with crystal violet. Stained cells on the bottom membrane were counted on five microscopic fields per sample. Graph data are means±s.d.; *P<0.05.

SVEC4-10 cell migration detected by wound-healing (or gap-closure) assay revealed dose-dependent suppression of wound closure up to 24 h after 2DG treatment, when the gap was completely filled with SVEC4-10 cells in the control group (Fig. 4A). Further study by Boyden chamber assay revealed that treatment of SVEC4-10 cells with 2DG for 24 and 48 h significantly inhibited cell migration compared with the control (Fig. 4B).

### 2DG suppresses formation of lamellipodia and filopodia and causes disorganization of F-actin filaments in murine endothelial cells

**Fig. 5.**
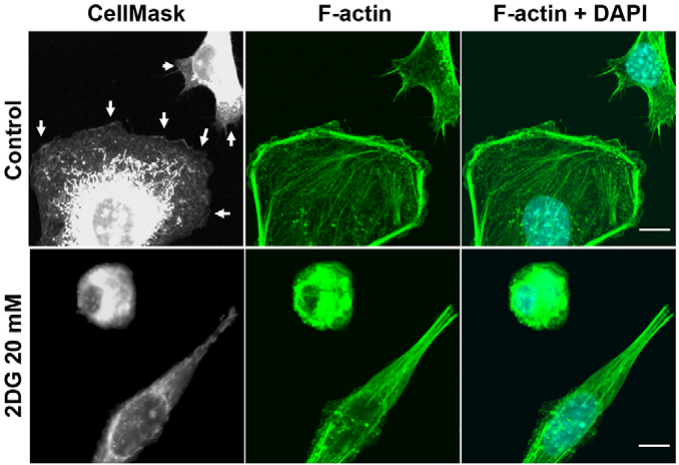
**2DG suppresses of lamellipodia and filopodia and causes disorganization of F-actin filaments in murine endothelial cells.** After 24 h of 2DG treatment, SVEC4-10 cells were stained with CellMask, F-actin and DAPI. Formation of lamellipodia (arrows in left column) was impaired and formation of filopodia (middle column) inhibited by 2DG treatment. Scale bars, 10 μm.

To study the microscopic structural changes of SVEC4-10 cells after treatment with 2DG, the SVEC4-10 cells were incubated with CellMask and fluorescein phalloidin. As shown in Fig. 5, treatment with 20 mM of 2DG up to 24 h resulted in remarkable impairment of formation of lamellipodia, resulting in smaller, shrunken cells compared with the normal cells, which had well-expanded gray-veil-like lamellipodia, by CellMask staining and laser confocal microscopic examination. When viewing cells treated with fluorescein phalloidin, which selectively labeled cytoskeletal F-actin, the 2DG-treated cells showed disorganized and prominently decreased intracellular F-actin, as well as blunted and less protruding filopodia from the cell surface, when compared with the control. The 2DG-treated cells became small and elongated.

### 2DG reduces the number of tumor vessels in both AS and DZ xenografts

**Fig. 6.**
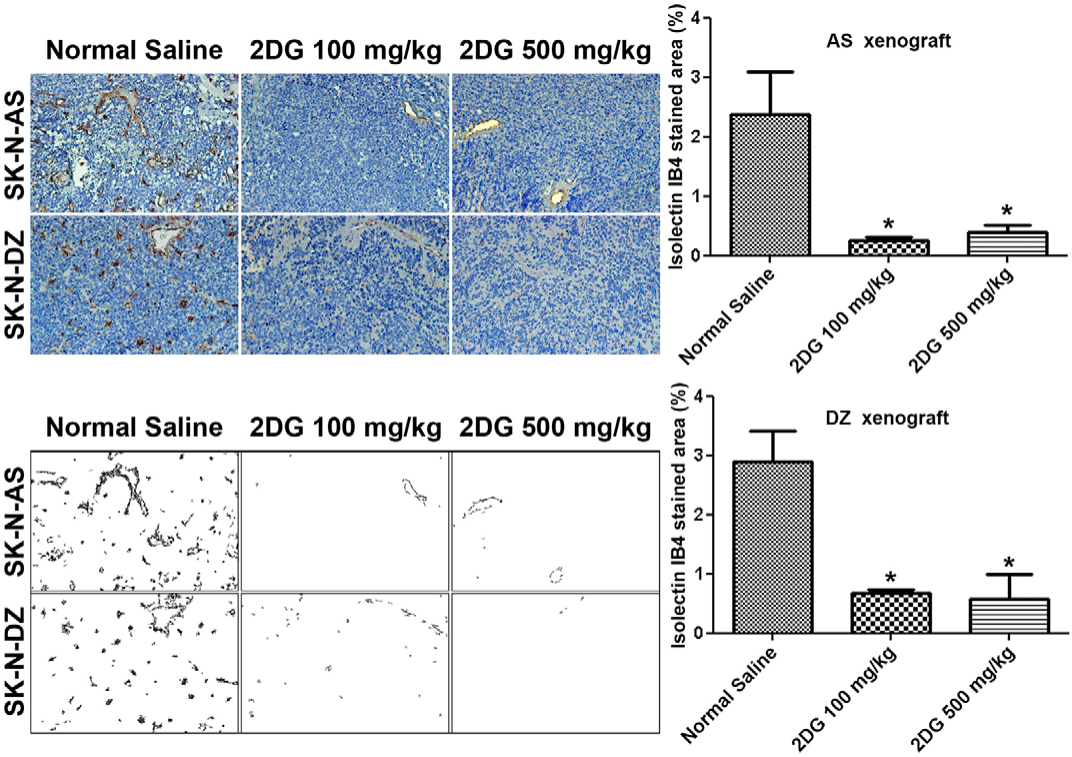
**2DG reduces the number of tumor vessels in both AS and DZ xenografts after six doses of treatment.** The slides with 3-µm-thick tissue sections of AS and DZ xenografts were stained with IB_4_ and counterstained with Hematoxylin (top panels). Five microscopic fields were taken per sample. The IB_4_-stained area was selected and highlighted by ImageJ (bottom panels) to determine the percentage of endothelial coverage in the xenograft. Graph data are means±s.e.m.; *P<0.05, *n*=5 for normal saline group and *n*=7 for 2DG groups (AS xenografts); *n*=5 for normal saline group and *n*=4 for 2DG groups (DZ xenografts).

To investigate whether 2DG causes a reduction in the number of tumor vessels in NB xenografts, we used isolectin IB_4_, which specifically labels endothelial cells. On the 15th day after injection of NB cells with three doses of 2DG treatment, there was a trend towards a decrease in the number of tumor vessels in both AS and DZ xenografts, compared with the control (supplementary material Fig. S3). On the 27th day after injection of NB cells, a significant reduction in the number of tumor vessels was shown in both AS and DZ xenografts in the group receiving either 100 or 500 mg/kg of 2DG for six doses (Fig. 6). Interestingly, 100 mg/kg of 2DG was effective in decreasing the number of blood vessels, and there was no dose-dependent advantage of 2DG treatment in this respect.

## DISCUSSION

This study discloses an unexpected finding that the glycolytic inhibitor 2DG is also effective to reduce the growth of AS xenograft in mice, which is comparable to DZ xenograft, particularly at a low dose. In our previously published *in vitro* study, DZ cells with *MYCN* amplification exhibited significantly higher HIF-1α expression and ATP production than AS cells without *MYCN* amplification, and were thus more responsive to 2DG, with significantly reduced cell growth and more cell apoptosis (Chuang et al., 2013). In the present study, we found that 2DG was equally effective to reduce HIF-1α and PDK1 expression in both DZ and AS xenografts. In AS xenograft, treatment with 2DG also resulted in significantly reduced c-Myc expression. However, in both DZ and AS xenografts, there was no significant increase of pro-apoptotic proteins, such as Bax and Bak. Ironically, the BH3-only pro-apoptotic protein Bad and p-Bad decreased after 2DG treatment in both DZ and AS xenografts. This finding implies that the *in vitro* effects of 2DG on NB cells can be applied only in certain respects to explain the findings in the mouse NB xenografts, regardless of whether it is from DZ or from AS cells.

It is known that metabolically active solid tumors are highly dependent on blood vessels to supply nutrients and to remove metabolic waste. 2DG has been shown to induce reactive oxygen species-triggered autophagy in endothelial cells or to inhibit angiogenesis in a transgenic retinoblastoma model at concentrations below those affecting tumor cells directly (Merchan et al., 2010; Wang et al., 2011). In this study, we have demonstrated that the mouse endothelial cell line SVEC4-10 is sensitive to 2DG treatment. 2DG not only inhibits proliferation and migration of SVEC4-10 cells, but also induces activation of caspase-3 and apoptosis in a dose-dependent manner. These studies underscore the importance of 2DG in tumor angiogenesis. It is not surprising that targeting of vascular sprouts could provide a new opportunity to improve anti-angiogenic therapy in cancer (Bergers and Hanahan, 2008; Rivera and Bergers, 2014).

We observed that 2DG inhibits the formation of lamellipodia and filopodia and causes derangement of F-actin, very similar to the effect of blockade of the glycolytic activator PFKFB3 (De Bock et al., 2013b). Lamellipodia and filopodia are responsible for cell migration, and inhibition of the formation of lamellipodia and filopodia by 2DG thus leads to suppression of migration and invasion (Mattila and Lappalainen, 2008; Ridley, 2011). Moreover, many cellular compartments, including the cytoskeleton, can sense homeostatic perturbations and translate them into a cell-death-initiating signal (Galluzzi et al., 2014). The disorganized F-actin in 2DG-treated SVEC4-10 cells is similar to death-associated protein kinase-induced cell death, indicating that the actin cytoskeleton is a major target for destruction during the process of apoptosis (Ivanovska et al., 2014; Leadsham et al., 2010). Consequently, there was a significant decrease in endothelial cells in both AS and DZ xenografts in mice treated with 2DG, leading to shrinkage of the tumor in this study, similar to that in the transgenic retinoblastoma model (Merchan et al., 2010).

In this study, we found that a low dose (100 mg/kg) of 2DG was as effective as a high dose (500 mg/kg) in both AS and DZ xenografts. The finding is different to our previous *in vitro* study, in which a low dose of 2DG (2 mM) was found to be as effective as a high dose (20 mM) only in DZ cells but not in AS cells (Chuang et al., 2013). However, the findings support the report that the effects of low doses of 2DG seem to affect actively forming capillaries preferentially, which was not demonstrated with other compounds (Merchan et al., 2010). It also supports the notion that tumor vessels are highly glycolytic and sensitive to metabolic manipulation, regardless of the underlying tumor (De Bock et al., 2013a,b).

In our previous *in vitro* studies, we have demonstrated decreased PDK1 expression and ATP contents and increased Bad expression in both AS and DZ cells treated with 2DG (Chuang et al., 2013). In the present study, we also found significantly decreased HIF-1α and PDK1 expression in both DZ and AS xenografts after treatment of the mice with 2DG for 3 weeks. Given that PDK1 participates in the metabolic switch of the cancer cells, targeting PDK1 has become a novel therapeutic option in some cancers (Fujiwara et al., 2013). Combination with other agents, such as epidermal growth factor receptor (EGFR), can cause regression of glioblastoma by reversing the Warburg effect (Velpula et al., 2013). Interestingly, HIF-1α was known to confer resistance to 2DG (Maher et al., 2007), which explained why 2DG induced upregulation of HIF-1α in AS xenografts in our *in vitro* studies (Chuang et al., 2013). However, the finding in the present *in vivo* study was different, and was more consistent with the report that HIF-1α can induce PDK3 (another isoform in the PDK family) and promotes a metabolic switch and drug resistance (Lu et al., 2008). The *in vivo* effects of 2DG might reverse the upregulation of both HIF-1α and PDK1.

Another unexpected finding in this study was the simultaneous downregulation of both Bad and p-Bad. Treatment with 2DG has been shown to increase expression of the pro-apoptotic BH3-only protein Bad in both AS and DZ cells in previous *in vitro* studies (Chuang et al., 2013). But this *in vitro* finding was challenged with recent reports that phosphorylation of BAD is essential for the survival of cancer stem cells, whereas ectopic expression of a phosphorylation-deficient mutant BAD induced apoptosis in cancer stem cells (Sastry et al., 2014). There was a positive correlation between overexpression of phospho-BAD and phosphorylated Akt in colorectal carcinoma (Khor et al., 2004). Phosphorylation of signal transducer and activator of transcription 1 and Bad reduces bortezomib-mediated apoptosis in cancer cells (Kao et al., 2013). The above findings indicate that Bad can be a marker of tumor progression and an attractive target for cancer therapy (Sastry et al., 2014), similar to our *in vivo* findings.

In AS xenografts, 2DG treatment resulted in downregulation of c-Myc. c-Myc is known to be one of the master regulators of cancer cell growth and metabolism (Miller et al., 2012). Targeting *MYC* oncogene and metabolic signaling partners offers new anti-cancer therapeutics (Teicher et al., 2012). Our findings are consistent with the above reports and echo the notion that expression of Myc oncoprotein could be a new prognostic factor for aggressive clinical behavior and thus deserves to be targeted (Wang et al., 2013, 2015).

In summary, the results of this study indicate a double advantage of the use of 2DG in treatment of mice loaded with either *MYCN*-amplified DZ cells or *MYCN*-non-amplified AS cells, by simultaneously targeting cancer cells and endothelial cells. This is unexpected from the previous *in vitro* study and provides a therapeutic opportunity to use 2DG or other glycolytic inhibitors in future treatment of patients with refractory NB.

## MATERIALS AND METHODS

### Neuroblastoma cell lines and endothelial cell line

Human NB cell lines, SK-N-AS and SK-N-DZ, were obtained from American Type Culture Collection (ATCC) and last tested on 15 April 2015. Tests were performed by using the Promega GenePrint10 System (GeneLabs Life Science Corp., Taipei, Taiwan) and analyzed with an ABI PRISM 3730 GENETIC ANALYZER and GeneMapper Software V3.7 (Applied Biosystems, Thermo Fisher Scientific Inc., Waltham, MA, USA). The report was 100% matched with short tandem repeat (STR) DNA profiles supplied by ATCC.

Mouse endothelial cell line SVEC4-10 was obtained from the Bioresource Collection and Research Center of the Food Industry Research and Development Institute, and experiments were performed within 6 months after cell resuscitation.

### Animal xenografts

Four-week-old male non-obese diabetic/SCID (NOD/SCID, NOD.CB17-Prkdc^scid^/NcrCrl) mice were purchased from the Ministry of Science and Technology, Taiwan. The care and the procedures were approved by the Animal Ethics Committee of the Kaohsiung Chang Gung Memorial Hospital. After 1 week of adaptation to the environment, the mice received subcutaneous injection of 1×10^7^ SK-N-DZ NB (DZ) cells or 5×10^6^ SK-N-AS NB (AS) cells into the right flank. Caliper measurement of tumor size was documented every 3 days. The tumor grew to about 4-6 mm in diameter by 1 week after implantation. The mice were randomized to receive intraperitoneal injection of normal saline or 100 or 500 mg/kg of 2DG (Sigma-Aldrich, St Louis, MO, USA) twice a week for 3 weeks, for a total of six doses. Eight mice from each group were killed on the second day of the last treatment, which was the 27th day after injection of NB cells. A subgroup of mice receiving intraperitoneal injection of three doses of normal saline (*n*=4 for DZ and *n*=3 for AS xenografts) or 100 mg/kg of 2DG (*n*=6 for both DZ and AS xenografts) was killed on the 15th day after injection of NB cells for the observation of the early effect of 2DG treatment. The tumor, liver, kidney and lung were harvested. The tumor harvested on the 27th day was weighed. A portion of the tissue was frozen in liquid nitrogen, and the remainder was either formalin fixed or placed in optimal cutting temperature compound (OCT) before freezing for future cryosectioning.

### Western blot analysis

Anti-PDK1, anti-HIF-1α, anti-c-Myc and anti-Bax antibodies were purchased from Cell Signaling Technology (Beverly, MA, USA) and anti-Bak antibody from Epitomics (Burlingame, CA, USA) for western blot analysis. The cryopreserved tumor tissues were homogenized and lysed. Protein lysates were prepared in a buffer containing complete protease inhibitor cocktail tablets (Roche Applied Science, Indianapolis, IN, USA). After lysis, the protein content was quantified using Bradford's method. Equal amounts of protein were loaded onto each lane of a polyacrylamide gel for SDS-PAGE. The proteins were transferred to Immobilon-P membranes (Millipore, Billerica, MA, USA) and incubated with 10% nonfat milk for 1 h. After three washes, the membranes were incubated with the indicated primary antibody overnight. After another three washes, the membranes were incubated with secondary horseradish peroxidase-conjugated goat anti-mouse antibodies (Millipore). The membranes were washed three times and visualized using an ECL system (GE Healthcare, Wauwatosa, WI, USA) with BioMax Light film (Kodak, Rochester, NY, USA). To quantify the signal intensity, Quantity One 1-D analysis software (Bio-Rad Laboratories, Hercules, CA, USA) was used.

### Immunohistochemistry

To study PDK1 expression in NB xenografts, silane-coated slides with 3-μm-thick sections of formalin-fixed, paraffin-embedded harvested xenografts were first treated with 3% hydrogen peroxide for 10 min, followed by microwave treatment for 10 min in 10 mM citrate buffer. The slides were then incubated with anti-PDK1 antibody (1:100 dilution; Abcam, Cambridge, UK) for 1 h. Sections probed with anti-PDK1 antibody were then incubated with a polymerized reporter enzyme staining system (ImmPRESS universal reagent, Vector Laboratories, Burlingame, CA, USA) for 30 min according to the manufacturer's recommendation. Finally, the signals were visualized by incubation with ImmPACT™ DAB Substrate (Vector Laboratories), counterstained with Mayer's Hematoxylin and mounted.

### Cell proliferation assay

Cell proliferation was detected by WST-1 assay, which was performed according to the manufacturer's instructions (Roche Applied Science, Mannheim, Germany). Briefly, 1×10^4^-2×10^4^ cells were plated in each well of a 96-well plate and cultured overnight. After treatment, the cells were incubated for another 24 or 48 h. The cell proliferation reagent (10 µl/well) was added, and the cells were incubated for another 2 h. The absorbance of the samples at 450 and 630 nm was measured using a 96-well spectrophotometric plate reader (Tecan, Mannedorf, Switzerland).

### Trypan Blue exclusion assay

After 2DG treatment, cells were collected and stained with 0.4% Trypan Blue (Gibco, Thermo Fisher Scientific Inc.) for 5 min. Under a microscope, live cells possess intact cell membranes that exclude Trypan Blue and express clear cytoplasm, whereas dead cells stain and present blue cytoplasm. The numbers of live cells and dead cells were counted, respectively, and calculated to obtain the ratio of dead cells.

### Flow cytometry to detect apoptosis

Apoptotic cell death was determined after staining with fluorescein isothiocyanate-conjugated annexin V and PI, according to the manufacturer's instructions (BD Pharmingen, BD Biosciences, San Jose, CA, USA). After treatment and collection, cells were resuspended in annexin V binding buffer and stained with annexin V/PI for 15 min. Data acquisition was carried out in a FACScalibur flow cytometer (BD Biosciences). Cells stained annexin V^+^, PI^−^ and annexin V^+^, PI^+^ were considered to be apoptotic.

### Cell migration detected by wound-healing assay and by Boyden chamber

For the wound-healing assay, cells were seeded in Culture-Insert (ibidi, Planegg/Martinsried, Germany) and cultured until the cells reached confluence. Culture-Insert was removed, and cells were washed with PBS to remove non-adherent cells. We then provided fresh medium containing 2DG (0, 2 or 20 mM) and photographed the plate at 0, 8 and 24 h to capture two different fields at each time point on each plate. The average wound width was measured between the two lines representative of cell migration determined by the mean of the furthest and the nearest cells at the leading edge.

For cell migration detected by Boyden chamber, cells were treated with 2DG (0, 2 or 20 mM) for 24 and 48 h, and then 5×10^4^ cells were suspended in growth medium without fetal bovine serum and seeded in the upper chambers of a 24-well transwell (8.0 µm; Millipore). Growth medium containing 20% fetal bovine serum as a chemoattractant was added in the lower chamber. After 8 h in culture, cells on the upper side of the filter were wiped out and cells on the bottom side of the filter were fixed with methanol and stained with 1% Crystal Violet solution overnight. Cells on the bottom side were subsequently counted under an inverted microscope. Results are expressed as the mean number of migrated cells in five random fields.

### Immunofluorescence and laser confocal microscopy

Mouse endothelial cells, SVEC4-10 (ATCC CRL-2181), 1×10^4^ cells per well, were inoculated in the chamber slide. The cultured cells were treated with 2DG for 24 h. After treatment, the samples were washed with PBS and fixed in 3.7% formaldehyde for 15 min, followed by an additional wash with PBS for 2 min three times, and then permeabilized with 0.1% Triton X-100 in PBS for 10 min. After permeabilization, the cell samples were blocked with 1% bovine serum albumin, followed by washing with PBS. The slides were then incubated with CellMask and fluorescein phalloidin (fluorescent phallotoxins, 1:100; Molecular Probes, Thermo Fisher Scientific Inc.) for 20 min, followed by washing with PBS and mounted in DAPI Fluoromount-G (SouthernBiotech, Birmingham, AL, USA). Photomicrographs were taken using a FluoView FV10i laser scanning confocal microscope (Olympus, Tokyo, Japan).

### Quantification of endothelial cell density after staining with isolectin IB_4_

To quantify the endothelial cell density in the xenograft, the formalin-fixed, paraffin-embedded sections of the tumors were stained with isolectin IB_4_-biotin conjugates (Molecular Probes). Briefly, after treatment with 3% hydrogen peroxide for 10 min followed by microwave treatment for 10 min in 10 mM citrate buffer, the section was incubated with isolectin IB_4_-biotin conjugates (1:200 dilution) for 30 min, then detected by a streptavidin-horseradish peroxidase product (BD Pharmingen) for 30 min and finally developed with DAB. After IB_4_ staining, the vascular areas (hot spots) with the highest density of endothelial cells were chosen and photographed for analysis with a US National Institutes of Health image-analysis software package (ImageJ). Five images (200× magnification) were chosen in each tumor section with care to avoid areas of necrosis. Color-discrimination thresholds were determined in the positively stained areas with a minimal threshold of 500 pixels. The endothelial cell density was calculated as the average of the IB_4_-positively stained areas in each tumor section (positively stained area pixels/total pixels).

### Statistical analysis

Statistical analyses were performed using SPSS software (version 12.0 for Windows; SPSS Inc., Chicago, IL, USA). For *in vivo* studies, including endothelial cell-density experiments, the data were analyzed using Tukey's honestly significant difference (HSD) post hoc tests. Data were expressed as the mean±s.e.m. For *in vitro* studies, all data presented in the figures are representative of at least three separate experiments and analyzed using Student's two-tailed *t*-test. Data were expressed as the mean±s.d. A *P*-value of less than 0.05 was taken to indicate statistical significance.

## Notes

This article is part of a subject collection on Model Systems in Drug Discovery: from Bench to Patient. See related articles in this collection at http://dmm.biologists.org/cgi/collection/drugdiscovery
